# An A2B Adenosine Receptor Agonist Promotes Th17 Autoimmune Responses in Experimental Autoimmune Uveitis (EAU) via Dendritic Cell Activation

**DOI:** 10.1371/journal.pone.0132348

**Published:** 2015-07-06

**Authors:** Mingjiazi Chen, Dongchun Liang, Aijun Zuo, Hui Shao, Henry J. Kaplan, Deming Sun

**Affiliations:** 1 Doheny Eye Institute and Department of Ophthalmology, University of California, Los Angeles, CA90033, United States of America; 2 Department of Ophthalmology and Visual Sciences, Kentucky Lions Eye Center, University of Louisville, Louisville, KY40202, United States of America; Ohio State University, UNITED STATES

## Abstract

We have recently reported that, although adenosine receptor (AR) agonists have a suppressive effect on Th1 autoreactive T cells, their effect on Th17 autoreactive T cells and γδ T cells is stimulatory and this effect is mainly mediated via A2A adenosine receptors (A2ARs). In this study, we further demonstrate that treatment of C57BL/6 (B6) mice with a selective A2B adenosine receptor (A2BR) agonist greatly enhanced the development of experimental autoimmune uveitis (EAU), whereas treatment with an A2BR antagonist significantly ameliorated severity of EAU. The A2BR agonist-treated mice showed augmented Th17, but not Th1, responses. Mechanistic studies showed that the A2BR agonist-induced enhancement of the Th17 response was significantly lower when TCR-δ^-/-^ mice received the same treatment and that transfer of γδ T cells into TCR-δ^-/-^ mice partially restored this effect. We also showed that dendritic cells (DCs) from A2BR agonist-treated mice showed a significantly increased ability to activate γδ T cells and Th17 autoreactive T cells. Thus, our previous studies have shown that, in EAU, activated γδ T cells possess greatly increased ability to enhance Th17 autoimmune responses. In the present study, we showed that exposure of DCs to A2BR agonist facilitated γδ T cell activation, leading to augmented Th17 responses and progressive EAU development. Our results further support our previous finding that AR agonists have distinct effects on Th1 and Th17 autoimmune responses.

## Introduction

Experimental autoimmune uveitis (EAU) is an animal model of T cell-mediated autoimmune disease that can be used to study the mechanism of induced autoimmune diseases in general and help develop therapeutic treatments [1–3]. Recent studies have shown that Th17 autoreactive T cells are the major pathogenic T cells in autoimmune diseases [4–8]. However, knowledge about the generation, differentiation, and activation of Th17 cells is still limited. We have previously demonstrated that the Th17 autoimmune response is determined by the pro- and anti-inflammatory effects of γδ T cells, which are controlled by their activation status [9–13]. In our search for molecules that affect γδ T cell activation, we examined the role of adenosine, as previous studies have shown that this small molecule affects the function of various immune cells, including lymphocytes [14–16], polymorphonuclear leukocytes [17,18], and macrophages/dendritic cells (DCs) [19–21].

Extracellular ATP, ADP, and adenosine are powerful signaling molecules and play an important role in controlling various patho-physiological responses, including inflammatory immune responses [22–24]. Large amounts of purines are released when tissue cells suffer damage during pathological conditions or when immune cells become activated [25,26]. Increased adenosine levels in the extracellular space are reported to decreased inflammation-induced tissue damage and injury [27,28], but high adenosine generation is also reported to undermine immune responses and enhance tissue damage [29,30]. These opposite effects of adenosine on inflammation suggest that control of adenosine receptor (AR) activation or inactivation using selective agonists and antagonists could have therapeutic implications in inflammation-related diseases [16,23,31,32].

In previous studies, we found that activation A2ARs has a strong regulatory effect on Th17 autoimmune responses [33,34]. Since there are four known AR subtypes (A1, A2A, A2B, and A3) that are expressed by various immune and non-immune cells, we wished to determine whether binding of adenosine to different ARs would induce a similar or different effect on the Th17 autoimmune response. In this study, we studied the effect of an A2BR agonist on Th1 and Th17 autoimmune responses and found that it had significantly enhanced development of EAU and that this effect was mainly due to its acting on Th17 autoreactive T cells. More importantly, A2BR antagonist treatment of mice undergoing EAU induction significantly ameliorated EAU. Our results support our previous finding [34] that AR agonists have distinct effects on Th1 and Th17 autoimmune responses.

## Materials and Methods

### Animals and reagents

Female C57BL/6 (B6), IFN-γ^-/-^, and TCR-δ^-/-^ mice on the B6 background, purchased from Jackson Laboratory (Bar Harbor, ME), were housed and maintained in the animal facilities of the University of California, Los Angeles and were used at 12–16 weeks of age. Experimental protocols were approved by the Institutional Animal Care and Use Committee of University of California Los Angeles (Protocol number: ARC#2014-029-03A). Recombinant murine IL-1 and IL-23 were purchased from R & D (Minneapolis, MN). Fluorescein isothiocyanate (FITC)-, phycoerythrin (PE)-, or allophycocyanin (APC)-conjugated mouse monoclonal antibodies (mAbs) against mouse CD73 (Clone TY/11.8), CD44 (Clone IM7), CD86 (clone GL-1), mouse MHC class II antigen (Clone: M5/114.15.2), αβ T cell receptor (TCR, clone H57-597), or γδ TCR (clone GL3) and isotype control antibodies were purchased from Biolegend (San Diego, CA). The selective A2BR agonist BAY 60–6538 and the selective A2BR antagonist MRS 1754 were purchased from Sigma-Aldrich (St. Louis, MO, USA) and were dissolved as a 1 mM stock solution in DMSO and diluted 1/10000 in culture medium before use.

### T cell preparation

CD3^+^ T cells were purified from TCR-δ^-/-^ or IFN-γ^-/-^ mice immunized with the human interphotoreceptor retinoid-binding protein (IRBP) peptide IRBP_1-20_ injected with A2BR agonist or vehicle, as described previously [9,11,13]. Briefly, nylon wool-enriched splenic T cells were sequentially incubated at 4°C for 10 min with FITC-conjugated anti-mouse CD3 mAb to isolate total responder T cells or with FITC-conjugated anti-γδ TCR mAb to isolate γδ T cells and for 15 min at 4°C with anti-FITC Microbeads (Miltenyi Biotec GmbH, Bergisch Gladbach, Germany), then the cells were separated into bound and non-bound fractions on an autoMACS separator column (Miltenyi Biotec GmbH). The purity of the isolated cells, determined by flow cytometric analysis using PE-conjugated Abs against αβ or γδ T cells, was >95%. The synthetic TLR agonist Pam3CSK (TLR2) was purchased from InvivoGen (San Diego, CA).

### EAU induction and evaluation

To induce EAU, B6 or IFN-γ^-/-^ mice were immunized subcutaneously at 6 spots at the tail base and on the flank with a total of 200 μl of emulsion consisting of 150 μg of peptide IRBP_1–20_ (amino acids 1–20 of human IRBP) (Sigma, St. Louis, MO) emulsified in complete Freund’s adjuvant (CFA) (Difco, Detroit, MI) and were injected i.p. with 200 ng of pertussis toxin (Sigma). The mice were then randomly grouped and injected i.p. with either diluted DMSO (vehicle), BAY 60–6583 (1.0 mg/kg) or MRS 1754 (2.0 mg/kg) on days 1, 4, 7, and 10 post-immunization and were examined three times a week till the end of the experiment (day 25 after immunization) for clinical signs of EAU by indirect fundoscopy; the pupils were dilated using 0.5% tropicamide and 1.25% phenylephrine hydrochloride ophthalmic solutions, and fundoscopic grading of disease was performed using the scoring system described previously [35]. For histology, whole eyes were collected at the end of the experiment and prepared for histopathological evaluation. The eyes were immersed for 1 h in 4% phosphate-buffered glutaraldehyde, then transferred to 10% phosphate-buffered formaldehyde until processed. Fixed and dehydrated tissues were embedded in methacrylate and 5 μm sections were cut through the pupillary-optic nerve plane and stained with hematoxylin and eosin.

### Assessment of Th1 and Th17 polarized responses

Responder CD3^+^ T cells (3 x 10^6^) prepared from IRBP_1-20_-immunized B6 or TCR-δ^-/-^ mice were co-cultured for 48 h with IRBP_1-20_ (10 μg/ml) and irradiated spleen cells (2 x 10^6^/well) as antigen-presenting cells (APCs) in a 12-well plate under either Th17 polarized conditions (culture medium supplemented with 10 ng/ml of IL-23) or Th1 polarized conditions (culture medium supplemented with 10 ng/ml of IL-12), then IL-17 and IFN-γ levels in the culture medium were measured using ELISA kits (R & D) and the number of antigen-specific T cells expressing IL-17 or IFN-γ determined by intracellular staining followed by FACS analysis, as described below.

### Cytoplasmic staining

The in vivo primed T cells were stimulated for 5 days in vitro with the immunizing antigen and APCs, then activated T cells were separated using Ficoll gradient centrifugation and stimulated in vitro for 4 h with 50 ng/ml of phorbol myristic acetate (PMA), 1 μg/ml of ionomycin, and 1 μg/ml of brefeldin A (Sigma, St. Louis, MO). The cells were then fixed, permeabilized overnight with Cytofix/Cytoperm buffer (eBioscience, San Diego, CA), intracellularly stained with Abs against IFN-γ or IL-17, and analyzed on a FACScalibur.

### Flow cytometry analysis

Aliquots of 2 x 10^5^ cells were double-stained with combinations of FITC- or PE-conjugated mAbs. Data collection and analysis were performed on a FACS_calibur_ flow cytometer using CellQuest software.

### Assessment of the enhancing effect of γδTCR^+^ T cells on Th17 autoreactive T cells

CD3^+^ T cells (1 x 10^6^) from immunized TCR-δ^-/-^ mice were stimulated with the immunizing peptide in 24-well plates for 5 days in the presence or absence of γδ T cells (5% of the total cell number) isolated from immunized B6 mice with or without A2BR agonist administration, then proliferating cells were separated on a Ficoll gradient, intracellularly stained with anti-IL-17 Abs, and subjected to FACS analysis.

### Generation of bone marrow dendritic cells (BMDCs)

BMDCs were generated by incubation of bone marrow cells for 5 days in the presence of 10 ng/ml of recombinant murine GM-CSF (R&D Systems), as described previously [36].

### Adenosine binding assay

γδ T cells seeded in 96-well cell culture plates at a density of 1×10^5^/ml in 100 μl of complete medium were incubated for 1 h with H^3^-adenosine at final concentrations of 0 to 12,000 nM in triplicate, then cell-bound and free H^3^-adenosine were separated by harvesting the cells on a cell harvester (Perkin Elmer) and the cell-associated radioactivity measured by liquid scintillation. Scatchard plot analysis was then performed and the dissociation constant and maximum binding capacity calculated.

### Statistical analysis

Experiments were repeated 4–5 times. Experimental groups were typically composed of six mice and the figures show the data from a representative experiment. The statistical significance of differences between the values for different groups was examined using Mann Whitney U-test.

## Results

### Injection of EAU-prone B6 mice with an A2BR agonist increases the severity of EAU and enhances the Th17 autoimmune response

**Fig 1:**
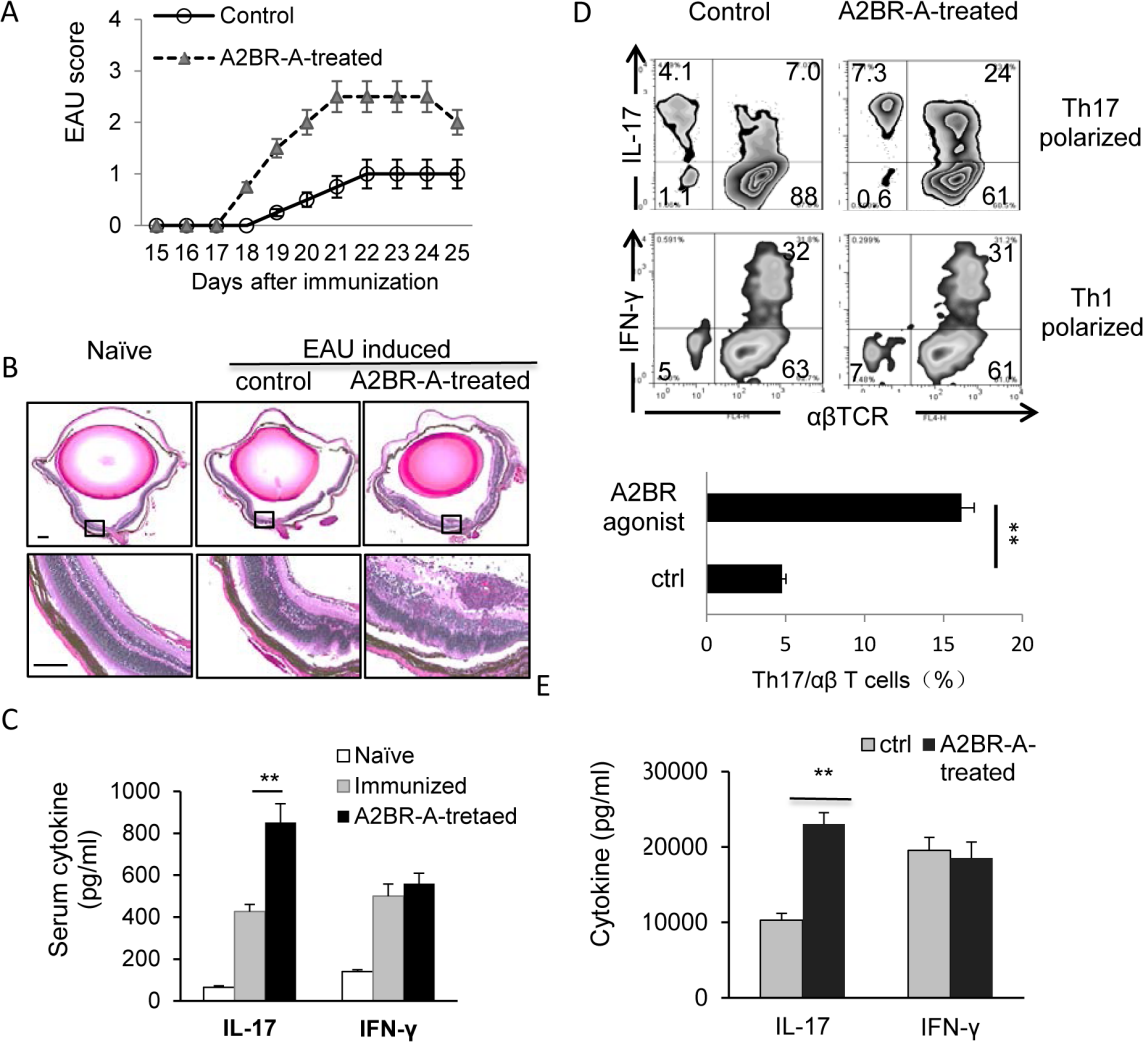
Administration of A2BR agonist to EAU-prone B6 mice enhanced EAU induction and activation of Th17 autoreactive T cells. (A&B). Two groups of B6 mice (n = 6) were immunized with IRBP1-20/CFA and injected i.p. with an A2BR agonist (A2BR-A, BAY 60–6583, 1 mg/kg) or vehicle (DMSO) on days 1, 4, 7, and 10 post-immunization. EAU was clinically scored by fundoscopy (A). On day 25 post-immunization, when the mice were sacrificed and the eye were taken and subjected to pathological examination (B). The H&E staining of the eye sections from naive, immunized but not BAY 60-6583-treated, and BAY60-6583-treated mice was shown. (C). Serum cytokine (IFN-γ and IL-17) levels measured by ELISA on day 13 post immunization. (D). Cytoplasmic staining assessing the percentage of IL-17^+^ cells among the in vitro antigen-stimulated IRBP-specific T cells. After 5 days’ of in vitro stimulation, the activated T cells were treated with PMA, ionomycin, and brefeldin, then were intracellularly stained with FITC-conjugated anti-TCR antibodies and PE-conjugated anti-IL-17 (upper panels)or anti-IFN-γ (lower panels) antibodies, followed by FACS analysis. (E). Determination of serum cytokine. 48-hour culture supernatants were assessed for IL-17 and IFN-γ by ELISA. Data are from one single experiment, which are representative of three independent experiments. **p< 0.01.

To determine the in vivo effect of an A2BR agonist on EAU development, randomly grouped B6 mice were immunized with a pathogenic dose of IRBP1-20, then received i.p. injections of the A2BR agonist BAY 60–6583 (1.0 mg/kg) or DMSO (vehicle) on days 1, 4, 7, and 10 post-immunization. EAU was monitored by fundoscopic examination for 15 days starting 10 days post-immunization and the mice were sacrificed at day 25 post-immunization and the eyes subjected to pathological examination. As shown in Fig 1A and 1B, the EAU peptide immunized mice treated with BAY 60–6583 showed significantly increased severity of ocular inflammation than those mice treated with vehicle. Fig 1C shows that serum IL-17 levels at day 13 post-immunization were markedly increased in immunized mice compared to naïve mice, and levels were twice as high in the BAY 60-6583-treated mice than the vehicle-treated mice. In contrast, serum IFN-γ levels were increased by immunization, but BAY 60–6583 had no effect (Fig 1C). To determine the effect of treatment on Th1 and Th17 autoimmune responses, CD3^+^ T cells from BAY 60-6583- or vehicle-treated immunized mice on day 13 post-immunization were prepared using a magnetic sorter and stimulated in vitro for 5 days with the immunizing peptide and APCs in the presence of IL-12 (Th1 polarizing conditions) or IL-23 (Th17 polarizing conditions), then the number of responding T cells expressing IL-17 or IFN-γ was assessed. As shown in Fig 1D, the percentage of IL-17^+^ T cells among T cells activated under Th17-polarizing conditions was significantly increased in BAY 60-6583-treated mice compared to control mice, whereas the percentage of IFN-γ^+^ T cells in the Th1 polarized T cells did not differ significantly between the two groups. Measurement of IL-17 and IFN-γ levels in the supernatants of T cells stimulated in vitro for 48 h with the immunizing peptide showed that T cells from BAY 60-6583-treated mice produced significantly higher levels of IL-17 than vehicle-treated mice, whereas similar IFN-γ production was seen in the two sets of cultures.

### Treatment of EAU-induced mice with an A2BR antagonist significantly ameliorates EAU

**Fig 2:**
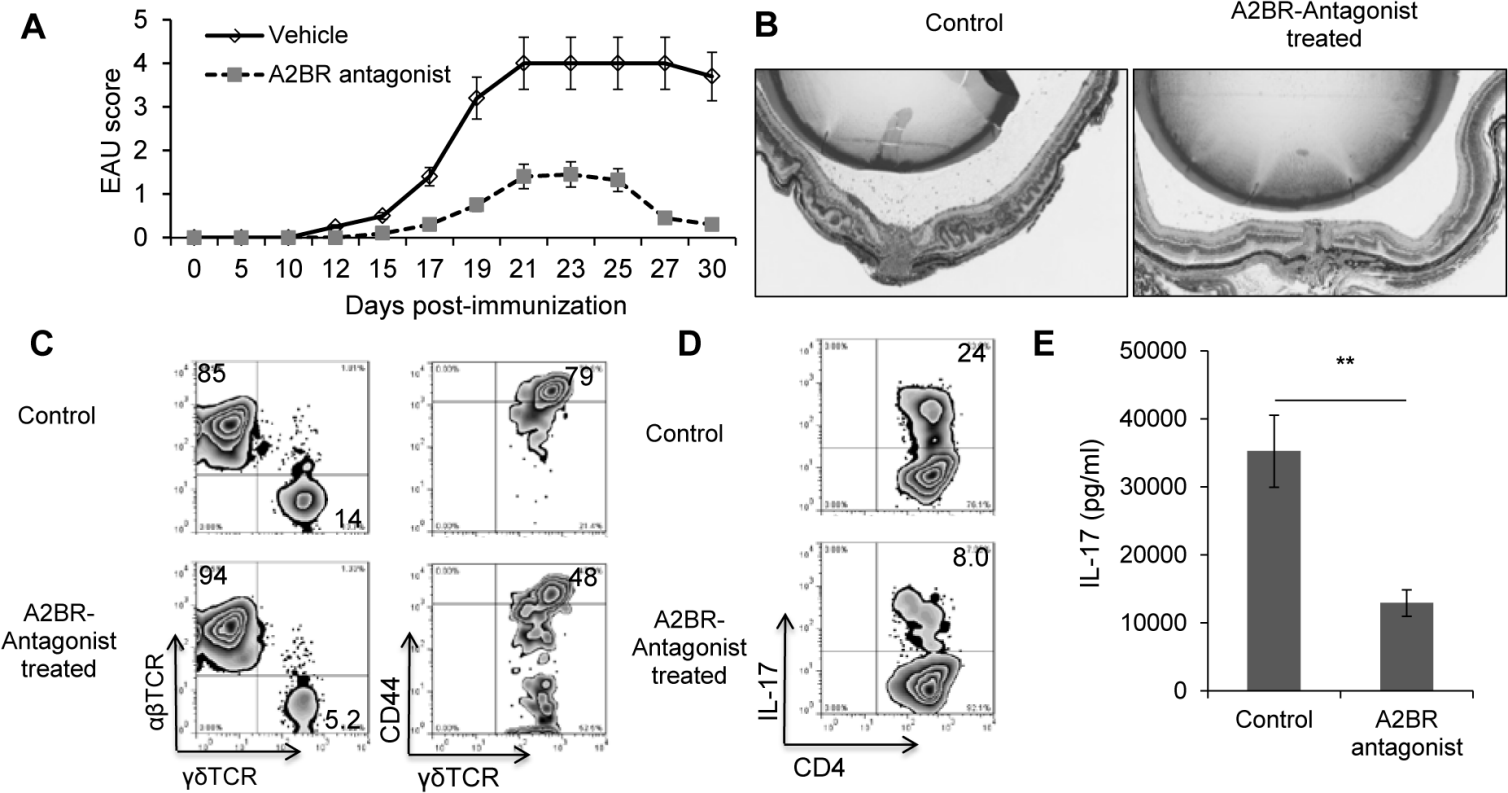
Administration of A2BR antagonist to IFN-γ^-/-^ mice mitigated the severity of induced EAU and the activation of Th17 autoreactive T cells. (A&B). IFN-γ^-/-^ mice were immunized with IRBP1-20/CFA alone or were also injected with an A2BR antagonist (MRS 1754, 2 mg/kg) via i.p., on days 1, 4, 7, and 10 post-immunization. The control mice received an injection of vehicle (diluted DMSO). EAU was scored by fundoscopy. All the mice were euthanized 30 days post-immunization, when the mice were sacrificed and the eye were taken and subjected to pathological examination. C) Surface staining assessing abundancy of γδ T cells among CD3^+^ splenic cells and CD44^+^ and CD44^-^ γδ T cells. D). Cytoplasmic staining assessing the percentage of IL-17^+^ cells among the in vitro antigen-stimulated IRBP-specific T cells, detailed as in Fig 1 legend. E). ELISA results from 48-hour culture supernatants were assessed for IL-17. Data are from one single experiment, which are representative of three independent experiments. **p< 0.01.

We then examined whether an A2BR antagonist would inhibit EAU by suppressing the development of Th17 T cell responses. As the average EAU score induced in vehicle-treated B6 mice was low and in the range of 0.5–1 (Fig 1), we used IFN-γ^-/-^ mice, which develop a stronger Th17 response using the same experimental protocol for EAU induction [37,38]. IFN-γ^-/-^ mice were immunized with a pathogenic dose of IRBP_1-20_ and were injected i.p. with either vehicle (DMSO) or the A2BR antagonist MRS 1754 (1.0 mg/kg) on days 1, 4, 7, and 10 post-immunization. As shown in Fig 2, the immunized IFN-γ^-/-^ mice treated with MRS 1754 showed a marked reduction in EAU score (Fig 2A) and developed significantly milder ocular inflammation than the vehicle-treated immunized mice (Fig 2B). In addition, as shown in Fig 2C, the number of γδ T cells in MRS 1754-treated mice was decreased compared to that in the vehicle-treated mice (left panels), as was their activation status, shown by expression of the activation marker CD44 (right panels). The number of IL-17^+^ T cells was also significantly decreased among CD4^+^ T cells from MRS 1754-treated mice compared to T cells from vehicle-treated mice (Fig 2D) and the culture supernatants of in vitro activated Th17 cells from MRS 1754-treated mice contained significantly lower amounts of IL-17 (Fig 2E).

### The Th17 response enhancing effect of the A2BR agonist is dramatically decreased in TCR-δ^-/-^ mice

**Fig 3:**
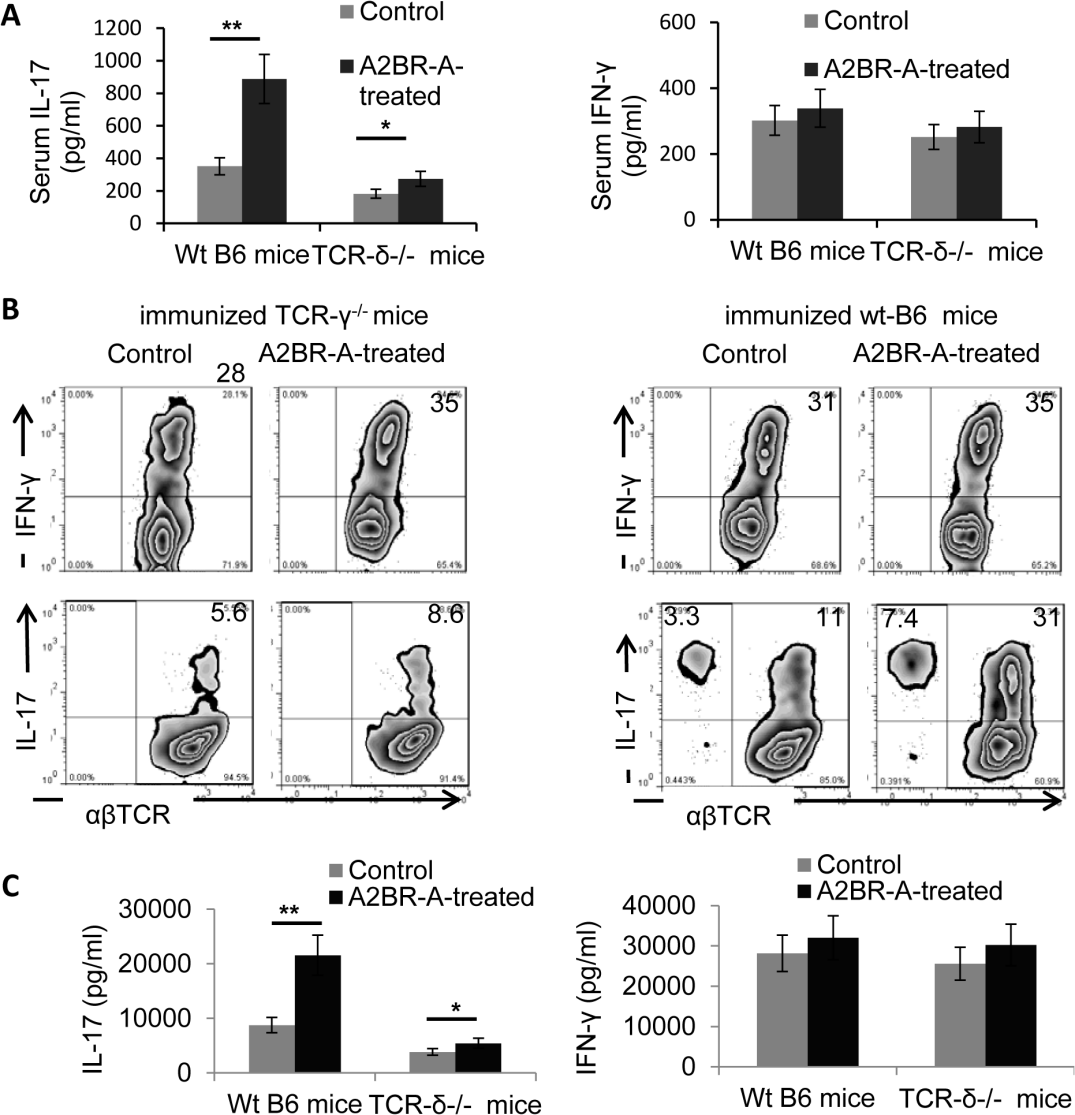
A2BR agonist failed to enhance Th17 response in TCR-δ^-/-^ mice. Groups of B6 and TCR-δ^-/-^ mice (n = 6) were immunized with IRBP1-20/CFA alone or were also injected with an A2BR agonist (A2BR-A, BAY60-6583, 1mg/kg) via i.p., on days 1, 4, 7, and 10 post-immunization. On Day 13 post immunization, serum cytokine (IFN-γ and IL-17) levels measured by ELISA (A). IL-17^+^ and IFN-γ^+^ cells among the proliferating T cells were assessed after 5-day in vitro stimulation of CD3^+^ T cells with the immunizing peptides and APCs under Th1 (Upper panel) and Th17 (lower panel) polarizing conditions (B) 48-hour culture supernatants of the cells cultured in (B) were assessed for IL-17 and IFN-γ by ELISA (C). Data are from one single experiment, which are representative of three independent experiments. *p<0.05.

As our previous studies showed that the enhanced Th17 response in EAU is associated with increased γδ T cell activation [10,13,39], we examined whether administration of the A2BR agonist BAY 60–6583 was able to enhance the Th17 response in γδ T cell-deficient mice using the same procedure described for B6 mice in Fig 1. As shown in Fig 3, the enhancing effect of BAY 60–6583 in TCR-δ^-/-^ mice was much lower than in B6 mice when assessed by serum IL-17 levels (Fig 3A), the number of IL-17^+^ T cells among activated IRBP-stimulated T cells (Fig 3B), or the amount of IL-17 secreted by in vitro activated T cells (Fig 3C).

### γδ T cells from A2BR agonist-treated B6 mice are functionally more activated

**Fig 4:**
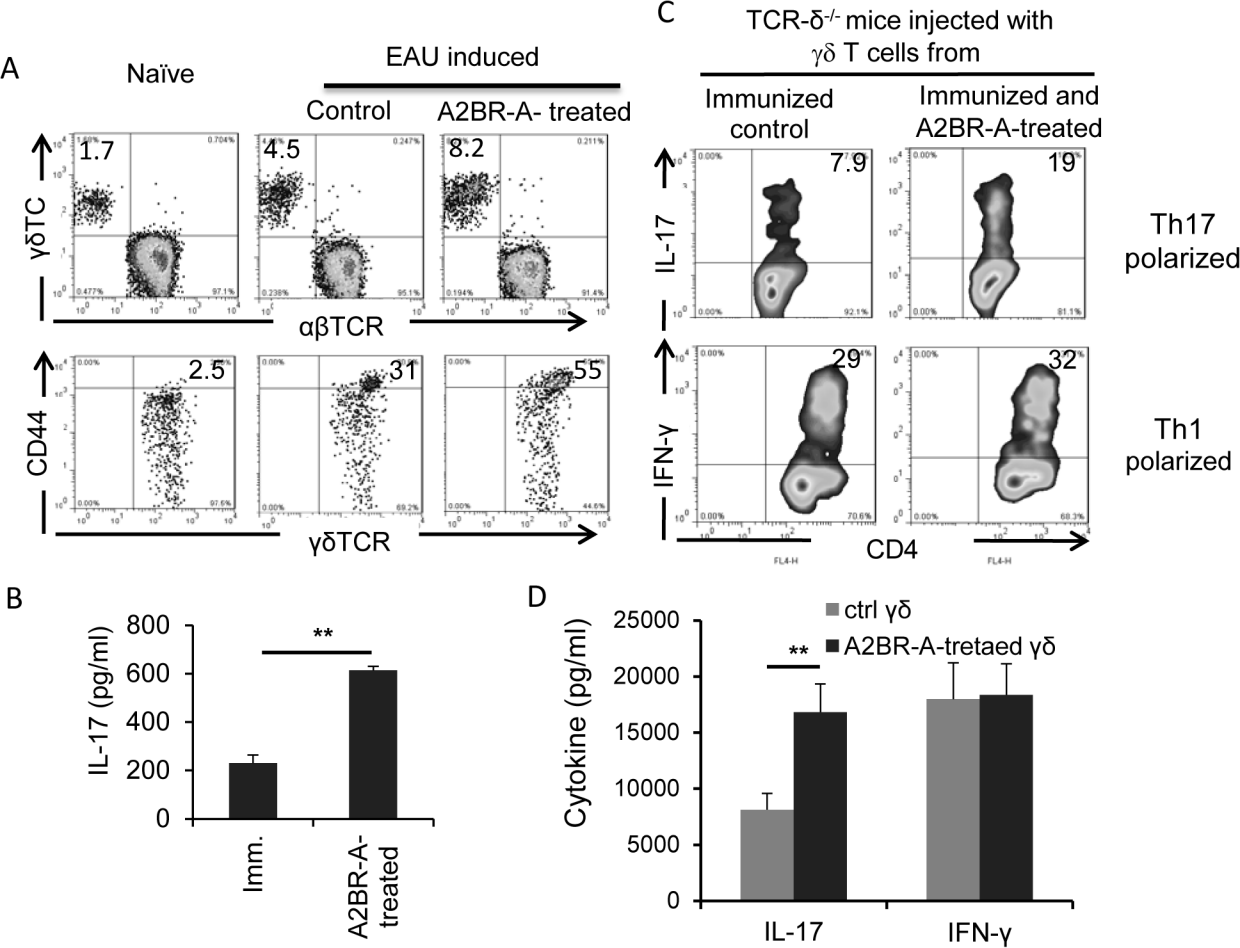
γδ T cells in A2BR agonist-treated mice were more activated in vivo and possesed increased Th17-promoting activity. A) Proportional numbers of γδ T cells and their activation status were compared among γδ T cells in naive, immunized, and BAY60-6538-treated, immunized mice. Splenic T cells were isolated from pooled mice, stained with anti-αβ, anti-γδ or CD44 antibodies, and followed FACS analysis. B) Cytokine production of freshly isolated γδ T cells was examined after cultured for 48 h in vitro, in the absence of additional stimulation. C) In vivo reconstitution study showing that γδ T cells isolated from BAY60-6538-treated mice have increased Th17-promoting activity when injected to TCR-δ^-/-^ mice. Groups of TCR-δ^-/-^ mice (n = 6) immunized with the uveitogenic peptide (IRBP1-20/CFA) were injected i.p. at the day of immunization with 10^5^ γδ T cells isolated from BAY60-6538-treated or untreated mice, before they were immunized with IRNP/CFA. 13 days post-immunization, splenic T cells were enriched and stimulated with immunizing peptide under Th1- and Th17-polarized conditions, and the activated T cells were stained for intracellular expression of IL-17 (top row) or IFN-g (bottom row). Results of one single experiment are shown, which were repeated for at least 3 times. D) 48-hour culture supernatants of the cells cultured in (C) were assessed for IL-17 and IFN-γ by ELISA. Data are from one single experiment, which are representative of three independent experiments **p<0.05.

We have previously shown that an enhanced Th17 response is closely associated with increased γδ T cell activation [10,11,13]. To determine whether γδ T cells in treated mice were activated and functionally more active, we compared the percentage of γδ T cells in total T cells from naïve B6 mice and immunized B6 mice with or without BAY 60–6538 treatment and examined them for expression of the T cell activation marker CD44, IL-17 production, and their ability to promote a Th17 response. The results showed that the percentage of γδ T cells increased significantly in immunized mice compared to naïve mice and increased further with BAY 60–6538 treatment (Fig 4A, upper panel). A significantly greater percentage (55%) of γδ T cells isolated from BAY 60-6538-treated mice expressed CD44, a cell surface marker of activated T cells, as compared to vehicle-treated mice (31%) (Fig 4A, lower panel). Moreover, freshly isolated γδ T cells from BAY 60-6538-treated mice secreted significantly higher amounts of IL-17 in the absence of in vitro stimulation, as compared to γδ T cells isolated from vehicle-treated immunized mice (Fig 4B). Based on our previous findings that T cells from immunized TCR-δ^-/-^ mice consistently generate low, but significant, numbers of IL-17^+^ T cells and that addition of γδ T cells from immunized B6 mice to pure responder T cells from immunized TCR-δ^-/-^ mice (a minimum of 2% of the total T cells) during an in vitro response greatly increases the activation of IL-17^+^ uveitogenic T cells [10,11,13], we examined whether γδ T cells isolated from BAY 60-6538-treated immunized B6 mice showed an increased Th17-promoting effect and found that γδ T cells from BAY 60-6538-treated mice showed a greater ability to increase IL-17 responses, as assessed by either the number of IL-17^+^ T cells among in vitro activated T cells (Fig 4C) or the amount of IL-17 in activated T cell culture supernatants (Fig 4D).

### BAY 60–6583 does not directly activate γδ T cells

**Fig 5:**
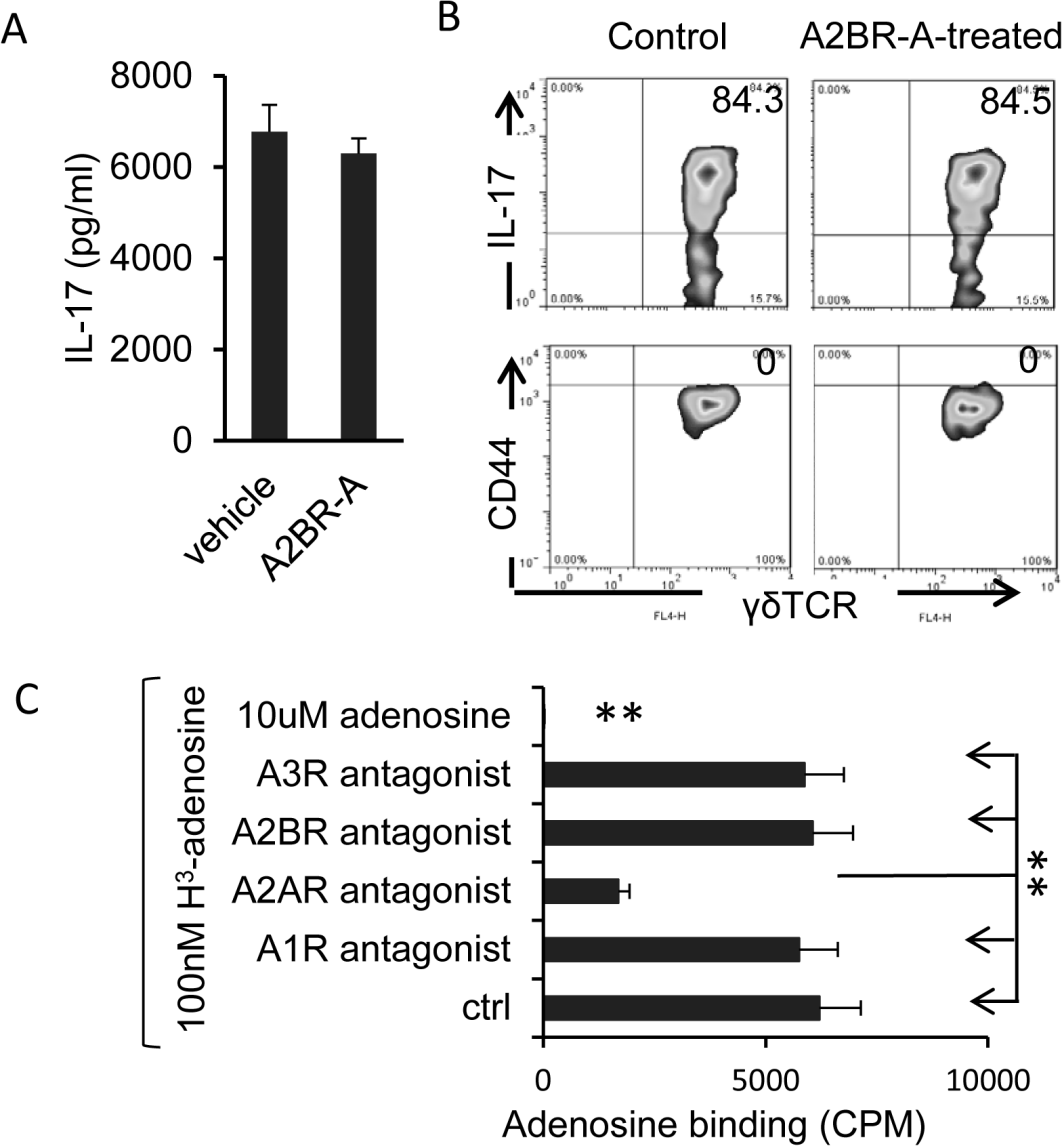
A2BR agonist does not directly stimulate γδ T cells. A and B) γδ T cells isolated from immunized B6 mice at day 13 post-immunization were cultured in the presence or absence of A2BR agonist for 48 h. then IL-17 levels in the supernatants were measured by ELISA (A) and expression of IL-17 and CD44 on the cells was examined by staining and FACS analysis (B). C) Adenosine binding assay showing that γδ T cells do not bind adenosine via A2BR. In 96-well plates, γδ T cells (1x10^5^/well) isolated from IRBP1-20-immunized mice at day 13 post-immunization were incubated for 30 min with vehicle (Control) or an antagonist of the A1AR (DPCPX, 50 nM), A2AR (SCH 58261, 100 nM), A2BR (MRS 1754, 100 nM), or A3AR (MRS1220, 5 μM), then for 1 h with radiolabeled adenosine (^3^H-adenosine, 100 nM). The amount of labeled adenosine bound was measured **P<0.01.

To determine whether BAY 60–6583 had a direct activating effect on γδ T cells, we isolated γδ T cells from immunized B6 mice and incubated them in vitro with and without BAY 60–6583 for 48 h, then measured cytokine levels in the culture supernatants and the expression of CD44 on the γδ T cells. The results showed that neither IL-17 production (Fig 5A) nor CD44 expression (Fig 5B) was significantly altered by exposure to BAY 60–6583. In addition, a competitive adenosine binding assay using AR subtype-specific antagonists revealed that γδ T cells did not bind adenosine via any ARs except A2AR and would therefore not bind BAY 60–6583 (Fig 5C).

### DCs acquire increased Th17-stimulating activity after exposure to BAY 60–6583

**Fig 6:**
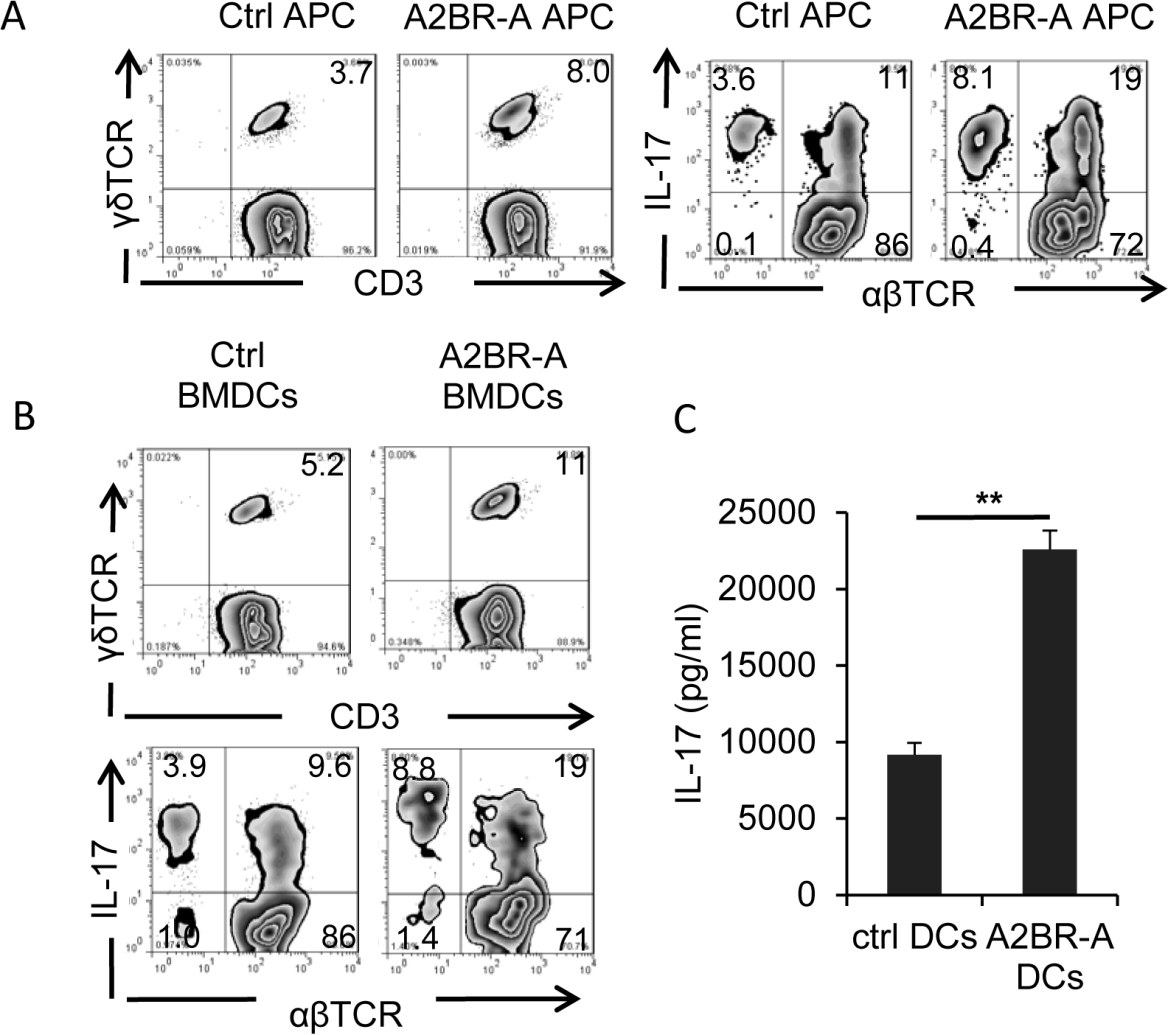
A2BR agonist-treated dendritic cells acquired increased ability to stimulate γδ and Th17 autoreactive T cells. A) Splenic APCs isolated from A2BR agonist-treated, immunized B6 mice have stronger ability stimulating γδ, and Th17 autoreactive T cells. Responder T cells were isolated from immunized B6 mice 13 days post immunization. After a 3-day co-incubation of T cells and splenic APCs, the activated T cells were separated by Ficoll gradient centrifugation and staining for γδ T cells or Th17 cells. B) A2BR agonist-treated BMDCs acquired increased stimulating activity to stimulate γδ, and Th17 autoreactive T cells. BMDCs were cultured from bone marrow cells of immunized B6 mice. After a 5 day co-culture of in vivo primed responder T cells with A2BR agonist-treated and-untreated BMDCs in the presence of immunizing peptide, the number of γδ T cells and IL-17^+^ T cells among activated T cells were examined (B) and the IL-17 and IFN-γ amounts in 48 h-stimulated culture supernatants were compared (C). **p< 0.01.

To determine how BAY 60–6583 enhanced the Th17 response without being able to direct activate γδ T cells, we examined whether this effect was mediated indirectly by stimulation of DCs. We therefore tested whether DCs from BAY 60-6583-treated mice had a greater stimulating activity on γδ T cells. First, we compared the stimulatory effect of splenic APCs from BAY 60-6583- or vehicle-treated immunized B6 mice using total splenic CD3^+^ T cells from immunized B6 mice as responder T cells to splenic APCs. After 3 days’ exposure in vitro to the splenic APCs, the numbers of γδ T cells among the responder T cells were assessed by FACS staining. As shown in Fig 6A, incubation with splenic APCs from BAY 60-6583-treated mice resulted in a higher percentage of γδ T cells in the CD3 T cells and the percentage of IL-17^+^ T cells in the αβ T cells than incubation with APCs from untreated mice. In addition, we prepared bone marrow dendritic cells (BMDCs) from bone marrow cells of immunized B6 mice by culturing them in GM-CSF-containing medium and examined whether cultured BMDCs showed a similar ability to activate γδ T cells and Th17 autoreactive T cells. Our results showed that, after in vitro exposure to BAY 60–6583, BMDCs showed a significantly increased ability to stimulate IL-17^+^ γδ T cells, as assessed by IL-17 production (Fig 6C) and, as shown in Fig 6B, to increase the percentage of γδ T cells in the CD3 T cells (upper panels) and the percentage of IL-17^+^ T cells in the αβ T cells (lower panels).

### The A2BR agonist stimulated BMDCs to produce increased amounts of IL-23 and IL-6

**Fig 7:**
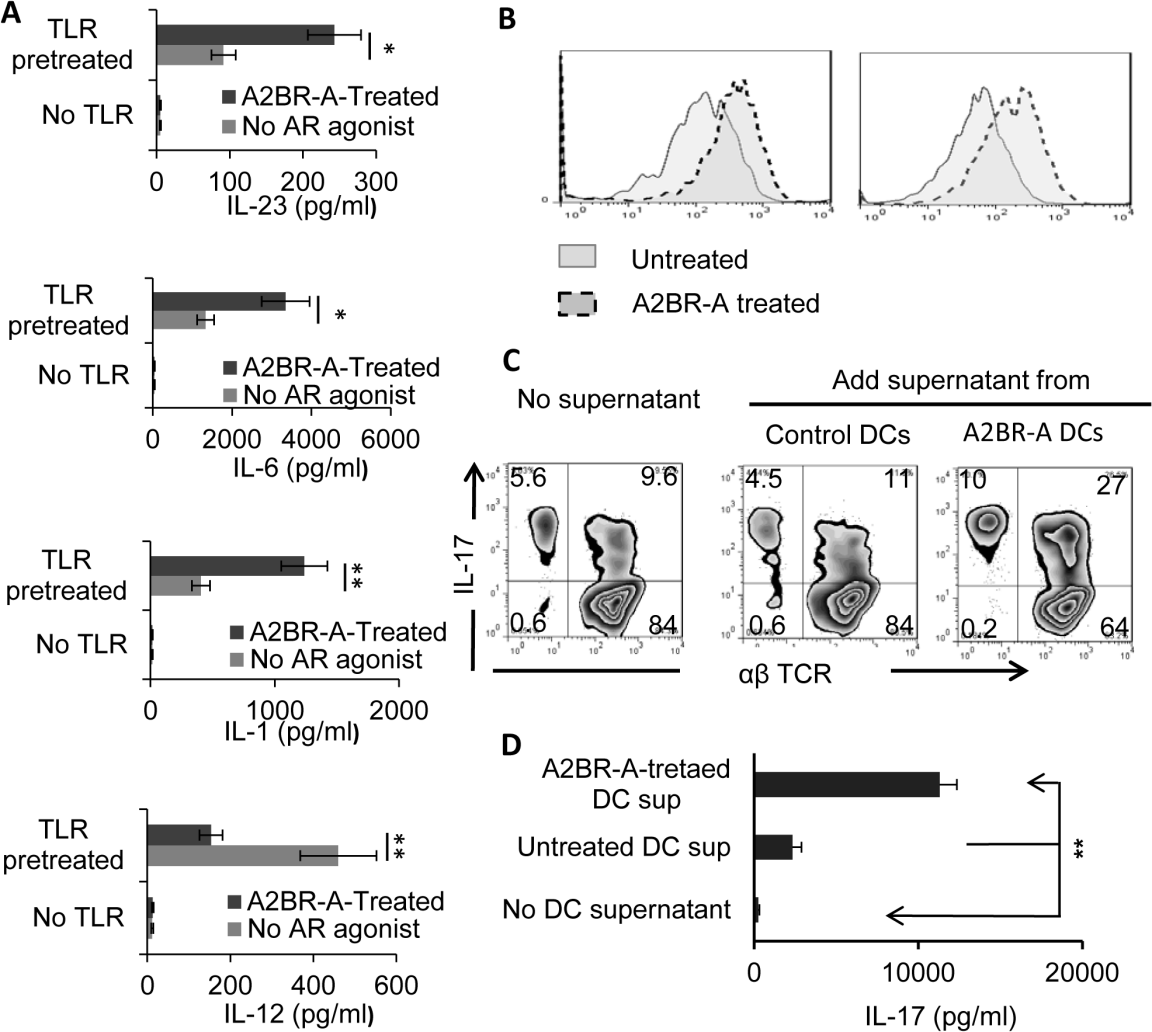
BMDCs incubated in vitro with the A2BR agonist produce increased amounts of IL-23 and IL-6 and have enhanced ability enhancing Th17 response. A) BMDCs cultured from immunized B6 mice were further cultured for 48h in the absence or presence of A2BR agonist (100 ng/ml), then IL-1, IL-12, IL-23 and IL-6 levels in the culture supernatants were measured by ELISA. B) Neither TLR2 ligand nor A2BR agonist along induced increased MHC II and CD86 expression on BMDCs, but a synergistic effect did. Cultured BMDCs were incubated with or without TLR2 ligand (Pam3CSK, 10μg/ml) and in the presence or absence of the A2BR agonist (100 ng/ml). 24h later, the expression of MHC II and CD86 on BMDCs was assessed by FACS after staining with FITC-anti-mouse MHC II or FITC-anti-mouse CD86 antibodies, respectively. C) The 48h supernatants of T cell-BMDC co-cultures described in Fig 7B were tested for IL-17 amount. **p<0.01. D) After an in vitro treatment with vehicle (control) or A2BR agonist for 24 h, the supernatants of BMDCs were taken and added to co-cultures of in vivo primed CD3^+^ responder T cells and splenic APCs, in the presence of the immunizing peptide and under Th17 polarizing conditions. After 5 days, proliferating T cells were stained intracellularly for IL-17.

Since DC-produced cytokines, such as IL-1, IL-6 and IL-23, are important promoters of the activation of Th17 autoreactive T cells [40–43], we examined whether the increased γδ T cell-stimulating effect of BMDCs from BAY 60-6583-treated immunized mice correlated with altered production of cytokines by BMDCs. When BMDCs from immunized B6 mice were incubated in vitro with BAY 60–6583 or vehicle, in the presence or absence of a Toll-like receptor 2 (TLR2) ligand (Pam3CSK), then levels of IL-1, IL-6, IL-12, and IL-23 in the culture supernatants were measured. Our results showed that treatment of TLR ligand induced BMDCs to produce all the four cytokines tested and BAY 60–6583 was unable to induce cytokine production from the BMDCs by itself. The levels of IL-1, IL-6, and IL-23 were markedly increased, but the IL-12 was significantly decreased in culture supernatants of BMDCs incubated with BAY 60–6583 and TLR ligand (Fig 7A). Neither TLR ligand along nor BAY 60–6583 induced increased expression of MHC class II and CD86 molecules on BMDCs, whereas when added together an enhanced expression was noticed (Fig 7B). In addition, when CD3^+^ T cells isolated from immunized B6 mice were stimulated by the immunizing antigen and APCs in the presence of supernatants from these BAY 60-6583-treated or vehicle-treated BMDCs, the former resulted in increased IL-17 secretion (Fig 7D) and an increased percentage of IL-17^+^ αβ T cells (Fig 7C).

## Discussion

Adenosine is a signaling molecule that is produced in high concentrations at sites of injured tissues [26,44–46]. Studies have shown that adenosine generation and consequent effects play a critical role in the pathophysiological changes of disease, particularly inflammatory diseases [22–24,31] and AR agonists and antagonists have been considering as promising pharmacological modulators of inflammation and immune responses [28,47–51].

Although extensive studies have been done examining the effect of AR agonists on immune response [17,23,24,31], studies examining the effect of AR agonists on immune responses have not adequately compared the effect on IFN-γ-producing (or Th1) and IL-17-producing (Th17) autoimmune responses, the major autoreactive T cells pathogenic to autoimmune diseases [4–8]. In addition, γδ T cell activation is the earliest pathogenic event in the development of induced EAU and our previous studies identified that aberrant γδ T cell activation is one of the key steps leading to an augmented Th17 response [9,10,33], the center of our hypothesis to test was that whether blockade of γδ T cell activation can prevent an enhanced Th17 response. In a previous report, we showed that γδ T cells express the highest levels of high affinity A2ARs, which allow them to bind adenosine effectively [33,34]. Moreover, A2AR ligation inhibited αβ T cell activation, but enhanced γδ T cell activation, leading to enhanced Th17 response [33,34]. Of particular interests, we have observed that the effects of AR agonist on Th1 and Th17 responses were completely dissociated, while AR agonists always had an inhibitory effect on the Th1 autoimmune response, their effect on the Th17 autoimmune response could be enhancing. Given that both Th1 and Th17 autoreactive T cells are pathogenic for autoimmune diseases [52–54], continue efforts to identify mechanisms by which adenosine enhances or inhibits should allow us to manage undesired effects of AR agonists on the Th17 autoimmune response and improve the therapeutic goal of controlling both Th1 and Th17 responses in autoimmune diseases. To examine whether main ARs other than A2ARs would have a similar or different effect in autoimmune development, we examined the role in EAU development of A2BR agonist, which has emerged as an important regulator of immune cell differentiation [31,55,56]. Our results show that administration to EAU-inducing B6 mice with an AR agonist specific for A2BR significantly enhanced the EAU development and treatment of EAU-inducing mice with an A2BR antagonist significantly ameliorated the induced EAU. Our results revealed that blockade of A2BR activation might limit the Th17-type autoimmune response and thus limit the disease susceptibility. Enhancing effect of A2BR agonist on Th17 autoimmune responses were also reported previously [16,32,57]. In previous study working on autoimmune encephalomyelitis [32], it has been shown that A2BR-specific antagonists alleviated the clinical symptoms of induced EAE; and A2BR-knockout mice developed less severe EAE. Our results additionally showed, however, the A2BR-mediated autoimmune enhancement is restricted to Th17, but not Th1, responses and both γδ T cells and DCs are crucially involved in the effector phases of the response. Thus, together with our previously reported results [33,34], we conclude that AR agonists have distinct effects on Th1 and Th17 autoimmune responses, regardless whether the effects are via A2AR or the A2BR.

Our study further supported the previous observation that AR agonists and antagonists are effective modulators in autoimmune encephalomyelitis [16,32], particularly the Th17-type autoimmune responses. Our study showed now that this applies to autoimmune uveitis. We additionally show that AR agonists have distinct effects on Th1 and Th17 autoimmune responses and that γδ T cells play a major role in the modulation of AR agonists on autoimmune responses.

We have emphasized on examining the mechanism of the Th17-enhancing effect. Indeed, the A2BR agonist effect on Th1 response could be more variable; conceivably, the treatment enhances the expression of the co-stimulatory factor on DC but suppressed Th1 response-required cytokines produced by DCs, typically the IL-12 (Fig 7). We believe the minimal difference on the conclusion on the role of A2BR in Th1 response could be due to the different approaches taken, in that the published papers [16,32] studied the effect of A2BR antagonist and they took a one injection treatment (day 10 post immunization); in contrast, we chose to inject A2BR agonist and used multiple injections (day 1,4,7 and 10 post immunization), in the considerations that administration of A2BR agonist is more interpretable because blockade of A2BRs using antagonist would damp the proinflammatory effect of DCs; in addition, decreased binding of adenosine by A2BR would enhance the ligation of A2ARs, which preferentially induces immunosuppression. As a result, A2BR antagonist showed a Th1 suppression effect. The in vivo effect of AR agonists is more sophisticated than we previously thought. In a previous paper we reported that injection of mice with a non-selective AR agonist, NECA, at an early stage after immunization had an inhibitory effect on both Th1 and Th17 responses, whereas injection of the same amount of NECA at a late stage, inhibited the Th1 response, but had an enhancing effect on the Th17 response [33]. Our observation supported the notion that the inflammatory environment has a strong impact on converting the effect of AR agonist from anti- to pro-inflammatory.

Our results also showed that although adenosine does not have a direct stimulating effect on γδ T cells, it strongly enhances the γδ-stimulating effect of proinflammatory cytokines and TLR ligands, which supports our previous argument that environmental factors play a major role modulating AR agonist effect in autoimmune response [33]. Identification of the mechanisms by which adenosine enhances or inhibits should allow us to improve the therapeutic goal of controlling both Th1 and Th17 responses in autoimmune diseases more effectively.

## Supporting Information

S1 ARRIVE Checklist The ARRIVE guidelines checklist. (PDF)
